# Deep Task-Based Quantization †

**DOI:** 10.3390/e23010104

**Published:** 2021-01-13

**Authors:** Nir Shlezinger, Yonina C. Eldar

**Affiliations:** 1 School of Electrical and Computer Engineering, Ben-Gurion University of the Negev, Beer-Sheva 8410501, Israel; 2 Faculty of Mathematics and Computer Science, Weizmann Institute of Science, Rehovot 7610001, Israel; yonina.eldar@weizmann.ac.il

**Keywords:** analog-to-digtal conversion, task-based quantization, deep learning

## Abstract

Quantizers play a critical role in digital signal processing systems. Recent works have shown that the performance of acquiring multiple analog signals using scalar analog-to-digital converters (ADCs) can be significantly improved by processing the signals prior to quantization. However, the design of such hybrid quantizers is quite complex, and their implementation requires complete knowledge of the statistical model of the analog signal. In this work we design data-driven task-oriented quantization systems with scalar ADCs, which determine their analog-to-digital mapping using deep learning tools. These mappings are designed to facilitate the task of recovering underlying information from the quantized signals. By using deep learning, we circumvent the need to explicitly recover the system model and to find the proper quantization rule for it. Our main target application is multiple-input multiple-output (MIMO) communication receivers, which simultaneously acquire a set of analog signals, and are commonly subject to constraints on the number of bits. Our results indicate that, in a MIMO channel estimation setup, the proposed deep task-bask quantizer is capable of approaching the optimal performance limits dictated by indirect rate-distortion theory, achievable using vector quantizers and requiring complete knowledge of the underlying statistical model. Furthermore, for a symbol detection scenario, it is demonstrated that the proposed approach can realize reliable bit-efficient hybrid MIMO receivers capable of setting their quantization rule in light of the task.

## 1. Introduction

Digital signal processing systems operate on finite-bit representation of continuous-amplitude physical signals. The mapping of an analog signal into a digital representation of a finite dictionary is referred to as *quantization* [1]. This representation is commonly selected to accurately match the quantized signal, in the sense of minimizing a distortion measure, such that the signal can be recovered with minimal error from the quantized measurements [2], ([3], Ch. 10). In many relevant scenarios, the task of the system is to recover some underlying parameters, and not to accurately represent the observed signal. In these cases, it was shown that by accounting for the system task in the design of the quantizers, namely by using *task-based quantization*, the accuracy in carrying out the task can be improved without increasing the number of bits used [4,5,6,7]. Such task-based quantization was shown to improve performance in channel estimation [8,9] and target identification in multiple-input multiple-output (MIMO) radar [10], when operating under tight bit budgets.

In practice, quantizers are typically implemented using analog-to-digital converter (ADCs), which operate on the input signal in a serial scalar manner. In such systems, the quantization rule is based on a uniform partition of a subspace of the real line, determined by the support of the quantizer. This quantization logic is very limited due to its simplicity: except for the specific case where the input is uniformly distributed over the support of the quantizer, uniform quantization is far from optimality ([11], Sec. 22) namely a more accurate representation can be obtained with the same number of bits. Furthermore, such quantizers typically do not account for the system task, namely they are *task-ignorant*. While the distortion induced by such inefficient quantization can be mitigated by assigning more bits for digital representation, i.e., using high-resolution quantizers, it can severely degrade the performance of bit-constrained systems.

Recent years have witnessed a growing interest in systems operating with low-resolution ADCs. In particular, the power consumption of ADCs typically grows with the bandwidth and the quantization resolution [12]. To maintain feasible cost and power usage when acquiring multiple signals at large frequency bands, low-resolution quantizers may be used. An example where such bit-constrained systems are popular is MIMO communication receivers, which simultaneously acquire and process multiple analog signals in order to recover the transmitted symbols and/or estimate the underlying channel, i.e., for a specific task. MIMO receivers operating at large spectral bands, e.g., millimeter wave systems [13], are commonly designed to acquire the channel output with low-resolution quantizers, and a large body of work focuses on schemes for carrying out the aforementioned tasks from coarsely discretized measurements, see, e.g., [14,15,16,17,18,19,20].

Quantizers are inherently non-linear systems. Hence, the design and implementation of practical quantizers which provide an accurate discrete representation while accounting for the system task, is difficult in general. Two notable challenges are associated with designing such task-based quantization systems: (1) In order to design the quantization scheme, one must have full knowledge of the stochastic model of the underlying signal [1,2], which may be unavailable in practice; (2) Even when the stochastic model is perfectly known, the scalar continuous-to-discrete rule which minimizes the representation error is generally unknown for most distributions under finite resolution quantization ([11], Ch. 23.1). A possible approach to tackle the second challenge is to use a uniform quantization rule, while applying additional processing in analog prior to quantization, resulting in an analog-digital hybrid system [21,22]. While such hybrid systems were shown to result in substantially improved performance for signal recovery tasks under bit constraints [5,6,7,9], their design is commonly restricted to a subset of analog mappings, e.g., linear processing [5,7]; and specific stochastic models, such as Gaussian observations [6,9]. Furthermore, these model-based quantization systems assume uniform quantizers, hence, they do not exploit the ability to use arbitrary quantization rules, while requiring accurate knowledge of the underlying statistical model.

An alternative approach to inferring the quantization system from the model, is to learn it from a set of training samples in a data-driven fashion. In particular, by using machine learning methods, one can implement task-based quantizers without the need to explicitly know the underlying model and to analytically derive the proper quantization rule. Existing works on deep learning for quantization typically focus on image compression [23,24,25,26,27], where the goal is to represent the analog image using a single quantization rule from which the image can be recovered and/or processed [28]. Alternatively, a large body of deep learning related works consider deep neural network (DNN) model compression [29,30,31], where a DNN operates with quantized instead of continuous weights. The work [32] used DNNs to compress and quantize high-dimensional channel state information in a massive MIMO feedback setup. The design of DNNs for processing one-bit quantized measurements in the digital domain, i.e., in the presence of task-ignorant quantizers, was considered for signal recovery in [33]; while DNN-based MIMO receivers with one-bit quantizers were studied in [34,35]. To the best of our knowledge, despite the importance of quantization with scalar ADCs in digital signal processing, the application of deep learning in such systems has not yet been studied.

In this paper, we consider the design of data-driven task-based quantizers, using scalar ADCs. Following [5,6], we propose a hybrid quantization system in which the analog mapping, the quantization rule, and the digital processing, are learned from training in an end-to-end fashion. The operation of the scalar ADCs is modeled as an intermediate activation layer. Unlike previous works which combined fixed uniform quantizers as part of a neural network [25,26,32], our method is specifically designed for learning scalar quantization mappings. We consider two generic tasks: estimating a set of parameters taking values in a continuous set from the quantized observations, and classifying the acquired signals. Our main target application is bit constrained MIMO receivers, in which these tasks may represent, for example, channel estimation and symbol detection, respectively.

Since continuous-to-discrete mappings applied in the quantization process are inherently non-differentiable, standard deep learning training algorithms, such as stochastic gradient descent (SGD), cannot be applied in a straight-forward manner. To overcome this difficulty, previous works used a simplified model of the quantizer, in which the quantization error is replaced by additive i.i.d. noise [25,26,32]. As the quantization error is a deterministic function of the analog input [36], the resulting model is relatively inaccurate, inducing a mismatch which, as we numerically demonstrate, degrades the ability to properly optimize the system in light of the task. Furthermore, this model is limited to fixed uniform continuous-to-discrete mappings, namely the quantization mapping cannot be learned during training. Here, we follow the soft-to-hard approach used in [23], approximating the continuous-to-discrete mapping during training with a differentiable one which faithfully represents the operation of the quantizer. This approach facilitates the application of back-propagation, while allowing learning the quantization mapping as part of an end-to-end network.

We numerically evaluate the performance of our proposed DNN-based system in MIMO communication scenarios. We first consider channel estimation, and compare our data-driven task-based quantizer to previous channel estimators from task-ignorant quantized measurements, as well as to the model-based task-based quantization system proposed in our previous work [5]. We also compare with the fundamental limits on channel estimation performance in MIMO systems with quantized observations, derived using indirect rate-distortion theory, which are achievable using optimal vector quantizers ([11], Ch. 23). Our results demonstrate that, even when the DNN-based quantizer is trained with samples taken from setups with different signal-to-noise ratio (SNR), it is still able to approach the performance of the optimal task-based quantizers with ADCs for varying SNRs, which is within a small gap of the fundamental performance limits.

Next, we test the data-driven quantizer for the task of symbol detection in multi-user MIMO communications. Here, we show that our quantizer achieves performance which is comparable to applying the maximum a-posteriori probability (MAP) rule without any quantization constraints, and is notably more robust to inaccurate channel state information (CSI). Furthermore, our deep task-based quantizer significantly outperforms the previously used approach of modeling quantization as additive noise during training, and we illustrate that the gap stems from the usage of a more accurate model for the quantization mapping. We also discuss how the proposed approach can be exploited to construct trainable task-based ADCs, by combining neuromorphic electronic systems [37] with digital neural networks, giving rise to robust, efficient, and accurate, data-driven methods for acquisition of analog signals.

The rest of this paper is organized as follows: Section 2 formulates the problem. Implementation of the data-driven task-based quantizer is presented in Section 3. Section 4 numerically evaluates the proposed quantizer in MIMO communication scenarios. Finally, Section 5 provides some concluding remarks.

Throughout the paper, we use boldface lower-case letters for vectors, e.g., x, and boldface upper-case letters for matrices, e.g., M. Sets are denoted with calligraphic letters, e.g., X. We use $I_{n}$ to represent the n×n identity matrix. Transpose, Euclidean norm, stochastic expectation, real part, and imaginary part are written as ${\left(\cdot \right)}^{T}$, $∥\cdot ∥$, E{·}, Re·, and Im·, respectively, R is the set of real numbers, and C is the set of complex numbers.

## 2. Preliminaries and Problem Statement

### 2.1. Preliminaries in Quantization Theory

To formulate the problem, we first briefly review the standard quantization setup. While parts of this review also appear in our previous work [5], it is included for completeness. We begin with the definition of a quantizer:

**Definition** **1 (Quantizer).** *A quantizer $Q_{M}^{n,k}\left(\cdot \right)$ with logM bits, input size n, input alphabet X, output size k, and output alphabet $\hat{X}$, consists of: (1) An encoding function $f_{n}:X^{n}\mapsto \left\{1,2,\ldots ,M\right\}≜M$ which maps the input into a discrete index. (2) A decoding function $g_{k}:M\mapsto {\hat{X}}^{k}$ which maps each index i∈M into a codeword $q_{i}\in {\hat{X}}^{k}$.*

We write the output of the quantizer with input $x\in X^{n}$ as $\hat{x}=g_{k}\left(f_{n}\left(x\right)\right)≜Q_{M}^{n,k}\left(x\right)$. *Scalar quantizers* operate on a scalar input, i.e., n=1 and X is a scalar space, while *vector quantizers* have a multivariate input. When the input size and the output size are equal, n=k, we write $Q_{M}^{n}\left(\cdot \right)≜Q_{M}^{n,n}\left(\cdot \right)$.

In the standard quantization problem, a $Q_{M}^{n}\left(\cdot \right)$ quantizer is designed to minimize some distortion measure $d:X^{n}\times {\hat{X}}^{n}\mapsto R^{+}$ between its input and its output. The performance of a quantizer is characterized using two measures: the quantization rate, defined as $R≜\frac{1}{n}\log M$, and the expected distortion $E\{d\left(x,\hat{x}\right)\}$. For a fixed input size *n* and codebook size *M*, the optimal quantizer is

(1) $$Q_{M}^{n,\mathrm{opt}}\left(\cdot \right)=\underset{Q_{M}^{n}\left(\cdot \right)}{\arg\min}E\left\{d\left(x,Q_{M}^{n}\left(x\right)\right)\right\}.$$

Characterizing the optimal quantizer via (1) and its trade-off between distortion and quantization rate is in general a very difficult task. Optimal quantizers are thus typically studied assuming either high quantization rate, i.e., R→∞, see, e.g., [38], or asymptotically large inputs, namely n→∞, commonly with i.i.d. inputs, via rate-distortion theory ([3], Ch. 10).

In *task-based quantization*, the design objective of the quantizer is some task other than minimizing the distortion between its input and output. In the following, we focus on the generic task of acquiring a random vector $s\in S^{k}\subseteq R^{k}$ from a statistically dependent random vector $x\in R^{n}$. The set S represents the possible values of the unknown vector: It can be continuous, representing an estimation task; discrete, for classification tasks; or binary, for detection tasks. This formulation accommodates a broad range of applications, including channel estimation and symbol detection, that are the common tasks considered in bit-constrained hybrid MIMO communications receivers [9], which are the main target systems considered in this work.

When quantizing for the task of estimation, under the objective of minimizing the MSE distortion, i.e., $d\left(s,\hat{s}\right)={∥s-\hat{s}∥}^{2}$, it was shown in [39] that the optimal quantizer applies vector quantization to the minimum mse (MMSE) estimate of the desired vector s from the observed vector x. While the optimal system uses vector quantization, the fact that such pre-quantization processing can improve the performance in estimation tasks was also demonstrated in [5], which considered scalar quantizers. However, it was also shown in [5,6] that the pre-quantization processing which is optimal with vector quantizers, i.e., recovery of the MMSE estimate of s from x, is no longer optimal when using scalar quantization, and that characterizing the optimal pre-quantization processing in such cases is very difficult in general. The fact that processing the observations in the analog domain is beneficial in task-based quantization motivates the hybrid system model which is the focus of the current work, and detailed in the following subsection. Due to the difficulty in analytically characterizing the optimal hybrid system, we consider a data-driven design, described in Section 3.

### 2.2. Problem Statement

As discussed in the introduction, practical digital signal processing systems typically obtain a digital representation of physical analog signals using scalar ADCs. Since in such systems, each continuous-amplitude sample is converted into a discrete representation using a single quantization rule, this operation can be modeled using *identical scalar quantizers*. In this work we study the implementation of task-based quantization systems with scalar ADCs in a data-driven fashion.

**Figure 1:**
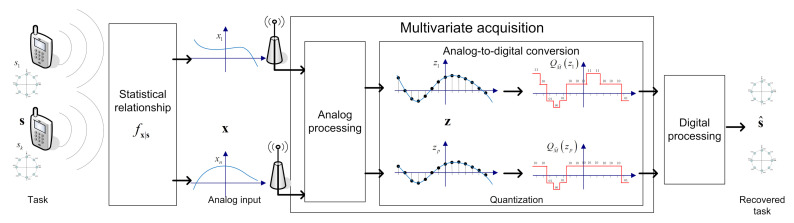
Hybrid task-based quantization system model. For illustration, the task is recovering a set of constellation symbols in uplink MIMO communications.

The considered signal acquisition system with scalar ADCs is modeled using the hybrid setup depicted in Figure 1, where a set of analog signals are converted to digital in order to extract some desired information from them. This model can represent, e.g., sensor arrays or MIMO receivers, and specializes the case of a single analog input signal. While acquiring a set of analog signals in digital hardware includes both sampling, i.e., continuous-to-discrete time conversion, as well as quantization, namely continuous-to-discrete amplitude mapping, we henceforth focus only the quantization aspect assuming a fixed sampling mechanism, and leave the data-driven design of the overall system for future investigation.

We consider the recovery of an unknown random vector $s\in S^{k}$ based on an observed vector $x\in R^{n}$ quantized with up to logM bits. The observed x is related to s via a conditional probability measure $f_{x|s}$, which is assumed to be unknown. For example, in a communications setup. the conditional probability measure $f_{x|s}$ encapsulates the noisy channel. The input to the ADC, denoted $z\in R^{p}$, where *p* denotes the number of scalar quantizers, is obtained from x using some pre-quantization mapping carried out in the analog domain. Then, z is quantized using an ADC modeled as *p* identical scalar quantizers with resolution $\tilde{M}≜\left\lfloor M^{1/p}\right\rfloor$. The overall number of bits is $p\cdot \log\tilde{M}\leq \log M$. The ADC output is processed in the digital domain to obtain the quantized representation $\hat{s}\in S^{k}$.

Our goal is to design a generic machine-learning based architecture for task-based quantization with scalar ADCs. The proposed system operates in a data-driven manner, namely it is capable of learning the analog transformation, quantization mapping, and digital processing, from a training data set, consisting of *t* independent realizations of s and x, denoted ${\left\{s^{\left(i\right)},x^{\left(i\right)}\right\}}_{i=1}^{t}$. In general, the training samples may be taken from a set of joint distributions, and not only from the true (unknown) joint distribution of s and x, as we consider in our numerical study in Section 4. We focus on two tasks which are relevant for MIMO receivers: An estimation task, in which S=R, representing, e.g., channel estimation; and classification, where S is a finite set, modeling, e.g., symbol detection. Our design is based on machine-learning methods, and specifically, on the application of DNNs.

## 3. Deep Task-Based Quantization

In the following, we present a deep task-based quantizer, which implements the system depicted in Figure 1 in a data-driven fashion using DNNs. To that aim, we first discuss the proposed network architecture in Section 3.1. Then, in Section 3.2 we elaborate on the discrete-to-continuous mapping and its training method, and provide a discussion on the resulting system in Section 3.3.

### 3.1. DNN Architecture

**Figure 2:**
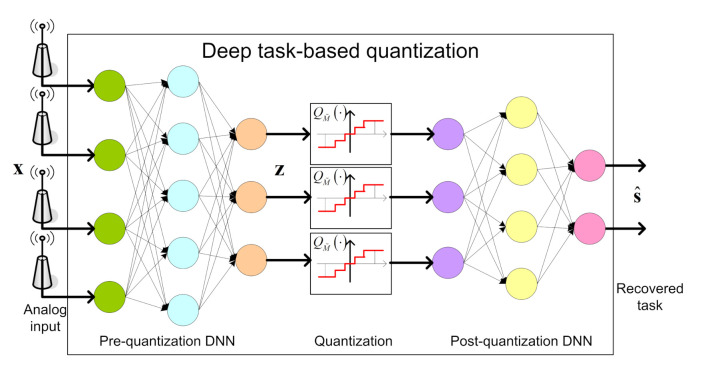
Deep task-based quantization system architecture.

We propose to implement a data-driven task-based quantizer using machine-learning methods. In particular, we realize the pre and post quantization mappings using dedicated DNNs, jointly trained in an end-to-end manner, as illustrated in Figure 2.

In the proposed architecture, the serial scalar ADC, which implements the continuous-to-discrete mapping, is modeled as an activation function between the two intermediate layers. The trainable parameters of this activation function determine the quantization rule, allowing it to be learned during training. The DNN structure cannot contain any skip connections between the multiple layers prior to quantization (analog domain) and those after quantization (digital domain), representing the fact that all analog values must be first quantized before processed in digital. The pre and post quantization networks are henceforth referred to as the *analog DNN* and the *digital DNN*, respectively. The system input is the n×1 observed vector x, and we use θ to denote the trainable parameters of the network. As detailed in Section 2.2, we consider two main types of tasks:

- Estimation: Here, the deep task-based quantizer should learn to recover a set of *k* unknown parameters taking values on a continuous set, i.e., S=R. By letting $\psi_{\theta }\left(\cdot \right)$ denote the mapping implemented by the overall system, the output is given by the k×1 vector $\hat{s}=\psi_{\theta }\left(x\right)$, which is used as a representation of the desired vector s. The loss function is the empirical MSE, given by
  (2) $$L\left(\theta \right)=\frac{1}{t}\sum_{j=1}^{t}{∥s^{\left(j\right)}-\psi_{\theta }\left(x^{\left(j\right)}\right)∥}_{2}^{2}.$$
- Classification: In such tasks, the deep task-based quantization should decide between a finite number of options based on its analog input. Here, S is a finite set, and we use |S| to denote its cardinality. The last layer of the digital DNN is a softmax layer, and thus the network mapping $\psi_{\theta }\left(\cdot \right)$ is a ${\left|S\right|}^{k}\times 1$ vector, whose entries represent the conditional probability for each different value of s given the input x. By letting $\psi_{\theta }\left(x;\alpha \right)$ be the output value corresponding to $\alpha \in S^{k}$, the decision is selected as the most probable one, i.e., $\hat{s}=\arg\max_{\alpha \in S^{k}}\psi_{\theta }\left(x;\alpha \right)$. The loss function is the empirical cross-entropy, given by
  (3) $$L\left(\theta \right)=\frac{1}{t}\sum_{j=1}^{t}-\log\psi_{\theta }\left(x^{\left(j\right)};s^{\left(j\right)}\right).$$

By using DNNs, we expect the resulting system to be able to approach the optimal achievable distortion for fixed quantization rate $R=\frac{1}{n}\log M$ and input size *n*, without requiring explicit knowledge of the underlying distribution $f_{x|s}$. Such performance is illustrated in the numerical example presented in Section 4.1.

The proposed architecture is generic, and its main novelty is in the introduction of the learned quantization layer, detailed in the following subsection. Our structure can thus be combined with existing dedicated networks, which are trainable in an end-to-end manner, as a form of transfer learning. For example, sliding bidirectional recursive neural networks (SBRNNs) were shown to achieve good performance for the task of symbol detection in non-quantized communication systems with long memory [40]. Consequently, one can design a deep symbol detector operating under quantization constraints, as common in, e.g., millimeter wave communications [13], by implementing the digital DNN of Figure 2 as an SBRNN. In this work we focus on fully connected analog and digital DNNs, and leave the analysis of combination with dedicated networks to future investigation.

### 3.2. Quantization Activation

Our proposed deep task-based quantizer implements scalar quantization as an intermediate activation in a joint analog-digital hybrid DNN. This layer converts its continuous-amplitude input into a discrete digital representation. The non-differentiable nature of such continuous-to-discrete mappings induces a major challenge in applying SGD for optimizing the trainable parameters of the network. In particular, quantization activation, which can be modeled as a superposition of step functions determining the continuous regions jointly mapped into a single value, nullifies the gradient of the cost function. Consequently, straight-forward application of SGD fails to properly set the pre-quantization network. To overcome this drawback, we first review the common approach, referred to henceforth as *passing gradient*. Then discuss how one can backpropagate via quantization mappings while faithfully accounting for its operation as well as enabling its optimization in the training procedure via *soft-to-hard quantization*.

#### 3.2.1. Passing Gradient

In this approach the quantized values are modeled as the analog values corrupted by mutually independent i.i.d. noise [25,26,32], and thus quantization does not affect the back-propagation procedure. Since the quantization error is deterministically determined by the analog value [36], the resulting model is quite inaccurate. Specifically, while under some input distributions, the quantization noise can be modeled as being *uncorrelated* with the input [36], they are not mutually independent. In fact, in order for the quantization error to be independent of the input, one should use substractive dithered quantization [41], which does not represent the operation of practical ADCs. Consequently, using this model for quantization during training results in a mismatch between the trained system and the tested one.

**Figure 3:**
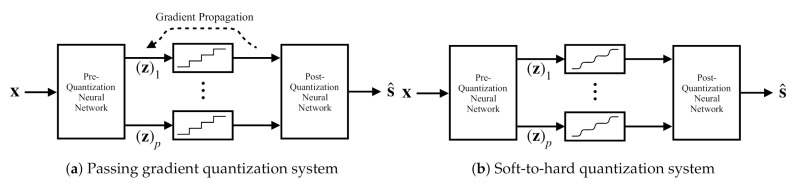
Task-based deep quantization architectures.

Under this model, the continuous-to-discrete mapping is fixed, representing, e.g., uniform quantization, and the training algorithm back-propagates the gradient value intact through the quantization layer. An illustration of this approach is depicted in Figure 3a. We expect the resulting system to obtain poor performance when non-negligible distortion is induced by the quantizers. In our numerical study presented in Section 4.2, it is illustrated that this method achieves relatively poor performance at low quantization rates, where scalar quantization induces an error term which is non-negligible and depends on the analog input. It is, therefore, desirable to formulate a network structure which accounts for the presence of scalar quantizers during training, and is not restricted to fixed uniform quantizers.

#### 3.2.2. Soft-to-Hard Quantization

An alternative approach is based on approximating the non-differentiable quantization mapping by a differentiable one. Here, we replace the continuous-to-discrete transformation with a non-linear activation function which has approximately the same behavior as the quantizer, as illustrated in Figure 3b. Specifically, we use a sum of shifted hyperbolic tangents, which are known to closely resemble step functions in the presence of large magnitude inputs. The resulting scalar quantization mapping is given by:

(4) $${\tilde{q}}_{\tilde{M}}\left(x\right)=\sum_{i=1}^{\tilde{M}-1}a_{i}\tanh\left(c_{i}\cdot x-b_{i}\right),$$

where $\left\{a_{i},b_{i},c_{i}\right\}$ are a set of real-valued parameters. Please note that as the parameters $\left\{c_{i}\right\}$ increase, the corresponding hyperbolic tangents approach step functions. Since we use a differentiable activation to approximate a set of non-differentiable functions [23], we refer to this method as *soft-to-hard quantization*.

In addition to learning the weights of the analog and digital DNNs, this soft-to-hard approach allows the network to learn its quantization activation function, and particularly, the best suitable constants $\left\{a_{i}\right\}$ (the amplitudes) and $\left\{b_{i}\right\}$ (the shifts). These tunable parameters are later used to determine the decision regions of the scalar quantizer, resulting in a learned quantization mapping. The parameters $\left\{c_{i}\right\}$, which essentially control the resemblance of (4) to an actual continuous-to-discrete mapping, do not reflect on the quantization decision regions (controlled by $\left\{b_{i}\right\}$) and their associated digital values (determined by $\left\{a_{i}\right\}$), and are thus not learned from training. The set $\left\{c_{i}\right\}$ can be either set according to the quantization resolution $\tilde{M}$, or alternatively, modified using annealing-based optimization [42], where $\left\{c_{i}\right\}$ are manually increased during training. The proposed optimization is achieved by including the parameters $\left\{a_{i},b_{i}\right\}$ as part of the network trainable parameters θ. Due to the differentiability of (4), one can now apply standard SGD to optimize the overall network, including the analog and digital DNNs as well as the quantization rule, in an end-to-end manner.

Once training is concluded, we replace the learned ${\tilde{q}}_{\tilde{M}}\left(\cdot \right)$ activation (4) with a scalar quantizer whose decision regions are dictated by the tunable parameters $\left\{a_{i},b_{i}\right\}$. In particular, since tanh(c·x−b)=0 for $x=\frac{b}{c}$, we use the set $\left\{\frac{b_{i}}{c_{i}}\right\}$ to determine the decision regions of the quantizer, and set the value of ${\tilde{q}}_{\tilde{M}}\left(x\right)$ at each decision region center as its corresponding representation level. Without loss of generality, we assume that $\frac{b_{0}}{c_{0}}\leq \frac{b_{1}}{c_{1}}\leq \ldots \leq \frac{b_{\tilde{M}-1}}{c_{\tilde{M}-1}}$ (when this conditions is not satisfied, the parameters are sorted and re-indexed accordingly). The resulting quantizer is given by

(5) $$Q_{\tilde{M}}^{1}\left(x\right)=\left\{\begin{array}{cc} -\sum_{i=1}^{\tilde{M}-1}a_{i} & x\leq \frac{b_{0}}{c_{0}}\\ {\tilde{q}}_{\tilde{M}}\left(\frac{b_{i}}{2c_{i}}+\frac{b_{i+1}}{2c_{i+1}}\right) & \frac{b_{i}}{c_{i}}<x\leq \frac{b_{i+1}}{c_{i+1}}\\ \sum_{i=1}^{\tilde{M}-1}a_{i} & \frac{b_{\tilde{M}-1}}{c_{\tilde{M}-1}}<x. \end{array}\right.$$

**Figure 4:**
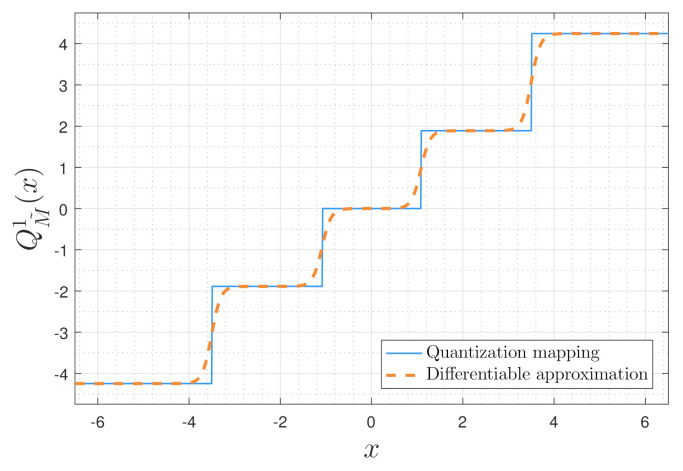
Soft-to-hard quantization rule illustration.

An illustration of how the differentiable mapping (4) is converted into a continuous-to-discrete quantization rule via (5) is depicted in Figure 4. The dashed smooth curve in Figure 4 represents the differentiable function after training is concluded, and the straight curve is the resulting scalar quantizer.

In the simulations study presented in Section 4.1, it is illustrated that the proposed method, which faithfully represents the presence of scalar quantizers during training and is capable of optimizing their decision regions, can outperform the model-based MSE minimizing task-based quantizer with scalar ADCs of [5], which requires complete knowledge of the underlying model, yet is restricted to uniform quantizers.

### 3.3. Discussion

The deep task-based quantizer proposed in Section 3.1 enables implementing quantization systems which learn how to map a continuous-amplitude input into a finite-bit representation in a manner which account for the task for which the signal is acquired. In the following we discuss some possible following directions which can facilitate transforming the proposed concept into DNN-aided task-based acquisition devices.

The system proposed in Section 3.1 implements hybrid multivariate acquisition using a set of identical scalar ADCs with learned decision regions, combined with DNN-based analog and digital transformations. While realizing DNNs in digital can be done in software, analog DNNs requires dedicated tunable hardware weights and activations. Such hardware networks, commonly referred to as neuromorphic electronic systems [37], implement configurable DNNs as analog components. Recent advances in memristors technology substantially facilitate the implementation of these hardware devices [43], contributing to the feasibility of our proposed deep task-based quantizer.

It is noted that in some applications, constrained analog structures may be preferable. For example, in MIMO receivers with a large number of antennas, i.e., massive MIMO, pre-quantization analog processing is commonly limited to phase shifting [21] or linear mappings [44]. Alternative hybrid acquisitions architectures for RF analog signals which include some level of controllable analog processing are dynamic metasurface antennas [45,46,47], which provide tunable analog combining in the form of Lorentzian filters. As such hybrid systems offer a limited range of analog mappings, one may prefer to use dedicated neural network circuitry for implementing deep task-based quantizers. In this case, the analog DNN is replaced with a single layer whose weights are restricted to have a unit magnitude, and this constraint has to be accounted for in training. Here we focus on generic analog DNNs, in which the weights are not constrained.

Our task-based quantizer can thus be implemented as a system consisting of adjustable analog hardware, configurable scalar quantizers, and software. The natural approach to set the parameters of the network would be to train the system model offline in software using an a priori acquired training set. The network weights and quantization decision regions obtained from this trained model can be then configured into the hardware components and the tunable ADCs, resulting in the desired task-based quantization system.

One can also envision an online trainable task-based quantizer, which is capable of further tuning its trainable parameters in real time to track dynamic environments, as in, e.g., [48,49,50]. For example, a communication receiver using a deep task-based quantizer for symbol detection, can exploit a priori knowledge of pilot sequences as labels corresponding to inputs acquired in real time. A major challenge in implementing such a system stems from the fact that both the labels $\left\{s^{\left(j\right)}\right\}$ as well as the inputs $\left\{x^{\left(j\right)}\right\}$ are required in order to update the network coefficients using conventional training algorithms, e.g., SGD. However, in our system the digital processor does not have direct access to the analog signal, but only to its quantized digital representation. Consequently, if the processor only uses digital values, it can only train the digital DNN using SGD. This challenge may be handled by allowing access to a high resolution quantized version of the analog signals, acquired in the specific time instances for which labels are available. An alternative approach is to use an error-correction-based update algorithm [51] instead of SGD, or reinforcement learning methods [52], since these techniques typically do not require direct access to the network input.

## 4. Application to MIMO Receivers

While the generic deep task-based quantizer proposed in Section 3 is applicable to a multitude of different setups, our main target application, studied in this section, is uplink multi-user MIMO communications. The problem of MIMO communications with low-resolution quantization is the focus of many recent works, including, e.g., [9,15,16,22,34]. Here, we consider a single cell multi-user MIMO system, in which $n_{u}$ single antenna users are served by a base station (BS) with $n_{t}$ antennas, which operates under quantization constraints. We focus on two tasks encountered in such setups: The first is channel estimation detailed in Section 4.1, for which we are capable of quantifying the performance gap of our system from optimality as well as comparing it to model-based designs. Then, in Section 4.2 we focus on symbol detection, which we treat as a classification task.

### 4.1. Channel Estimation Task

We first consider channel recovery, which is an estimation task commonly encountered in MIMO systems. We focus on a specific scenario for which we can compute both the fundamental performance limits, namely a lower bound on the achievable recovery accuracy which holds for any bit constrained system, as well as the performance of the best hybrid system restricted to using linear operations and uniform quantization, derived in [5]. These performance measures, which correspond to model-based systems, are used as a basis for comparison to evaluate our proposed data-driven task-based quantizer. The main motivation for the study detailed in this subsection is thus to compare the performance achievable using our proposed deep task-based quantizer to model-based techniques and the fundamental performance limits in a specific scenario where these values are computable.

In the following, we consider a channel estimation task carried out in a time diversity duplexing manner as in [9], using orthogonal pilot sequences of length $\tau_{p}\geq n_{u}$. We use $\Phi \in C^{\tau_{p}\times n_{u}}$ to denote the known pilot sequence matrix, where the orthogonality of the pilots implies that $\Phi^{H}\Phi =\tau_{p}\cdot I_{n_{u}}$, and *P* is the SNR. Additionally, let $h\in C^{n_{u}\cdot n_{t}}$ be a random vector whose entires are i.i.d. zero-mean unit-variance complex normal channel coefficients, and $w\in C^{\tau_{p}\cdot n_{t}}$ be a random vector with i.i.d. zero-mean unit-variance complex normal entries mutually independent of h, representing the additive noise at the BS. The observed signal $y\in C^{\tau_{p}\cdot n_{t}}$, used by the BS to estimate h, can be written as ([15], Equation (4)):

(6) $$y=\sqrt{P}\left(\Phi ⊗I_{n_{t}}\right)h+w,$$

where ⊗ is the Kronecker product.

To put the setup in (6) in the framework of our problem formulation, which considers real-valued signals, we write the observations as x=ReyT,ImyTT and the unknown channel as s=RehT,ImhTT. Consequently, the number of measurements is $n=2\cdot \tau_{p}\cdot n_{t}$, the number of unknown parameters is $k=2\cdot n_{u}\cdot n_{t}$, and their ratio is $\rho =\frac{\tau_{p}}{n_{u}}$, which is not smaller than one.

The performance measure for evaluating the quantization systems here is the average MSE, namely $\eta =\frac{1}{k}E\left\{{∥s\;-\;\hat{s}∥}^{2}\right\}$. For the above model, the average MMSE, which is the optimal performance achievable with no quantization constraints, is given by $\tilde{\eta }=\frac{1}{2(1+P\cdot \tau_{p})}$. In the presence of quantization constraints, the optimal approach is to quantize the MMSE estimate [39], and the resulting average distortion is obtained from rate-distortion theory ([3], Ch. 10.3) as

(7) $$\eta_{\mathrm{opt}}=\tilde{\eta }+\frac{P\cdot \tau_{p}}{2(1+P\cdot \tau_{p})}2^{-2\rho \cdot R}.$$

Please note that $\eta_{\mathrm{opt}}$ is achievable using optimal vector quantization in the limit $n_{t}\to \infty$. For finite $n_{t}$ and scalar quantizers, (7) serves as a lower bound on the achievable performance. We thus refer to $\eta_{\mathrm{opt}}$ as the *fundamental performance limit*.

We now numerically evaluate our proposed deep task-based quantizer, compared to the fundamental performance limit in (7), as well as to the performance of the task-based quantizer with scalar uniform ADCs designed in [5], denoted $\eta_{\mathrm{sc}}$. It is noted that while our proposed system can modify the quantization regions, the model of [5] assumes fixed uniform quantizers. Consequently, the average MSE of the system of [5] does not necessarily lower bound the performance of our proposed system. We also note that the system of [5] requires full knowledge of the underlying statistical model, namely the SNR as well as the distribution of h and w.

We simulate a multi-user MIMO network in which a BS equipped with $n_{t}=10$ antennas serves $n_{u}=4$ users. We set the SNR to be P=4 and the number of pilots to $\tau_{p}=12$. As in [15], we fix the pilots matrix Φ to be the first $n_{u}$ columns of the $\tau_{p}\times \tau_{p}$ discrete Fourier transform matrix. In the implementation of the deep quantizers, we set the pre and post quantization DNNs to consist of linear layers. The motivation for using linear layers stems from the fact that for the considered setup, the MMSE estimate is a linear function of the observations. Furthermore, this setting guarantees fair comparison with the model-based system of [5], which focused on linear analog and digital processing. Following ([5], Cor. 1), we evaluate the average MSE of our proposed systems with p=k quantizers. We consider two training sets, both of size $t=2^{15}$: In the first training set, representing *optimal training*, the realizations ${\left\{s^{\left(i\right)},x^{\left(i\right)}\right\}}_{i=1}^{t}$ are sampled from the true joint distribution of s,x; In the second training set, representing *SNR uncertainty*, ${\left\{s^{\left(i\right)},x^{\left(i\right)}\right\}}_{i=1}^{t}$ are sampled from the joint distribution of s,x with different values of *P*, uniformly randomized over the set [1,10] for each realization. At the end of the training session, we fix the quantizer to implement the continuous-to-discrete rule in (5). We numerically evaluate our trained proposed deep quantizer using $2^{10}$ independent channel realizations.

**Figure 5:**
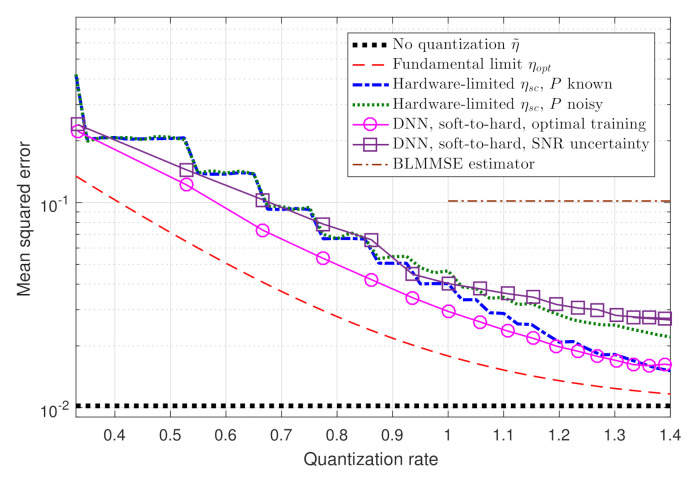
Numerical MSE versus theoretical measures.

In Figure 5 we depict the resulting performance versus the quantization rate $R=\frac{1}{n}\log M$ in the range R∈[0.33,1.4]. The empirical performance is compared to three theoretical measures: the MMSE $\tilde{\eta }$; the fundamental performance limits of channel estimation from quantized measurements, given by $\eta_{\mathrm{opt}}$ in (7); and the performance of the analytically derived task-based quantizer with scalar ADCs [5], denoted $\eta_{\mathrm{sc}}$. Since [5] requires perfect knowledge of the underlying model, and particularly of the SNR *P*, and as this information may not be available accurately in practice, we also consider the case where [5] uses an estimation of *P* corrupted by zero-mean Gaussian noise with variance $\frac{P}{4}$. Finally, we compute the average MSE of the BLMMSE estimator proposed in [15] via ([15], Equation (15)). Since the BLMMSE estimator quantizes the observed signal without analog pre-processing, it is applicable only for R≥1.

Observing Figure 5, we note that the performance of our soft-to-hard deep quantizer is within a relatively small gap of the fundamental performance limits. Furthermore, the fact that the soft-to-hard method is not restricted to uniform quantizers allows it to outperform the model-based $\eta_{\mathrm{sc}}$, especially in lower quantization rates. Finally, we note that in the presence of SNR uncertainty, the performance of the soft-to-hard method is similar to $\eta_{\mathrm{sc}}$ with noisy SNR estimate, and that both outperform the BLMMSE estimator of [15]. This indicates that our proposed scheme is applicable also when the training data is not generated from the exact same distribution as the test data. Our results demonstrate the ability of deep task-based quantization to implement a feasible and optimal-approaching quantization system in a data-driven fashion.

### 4.2. Symbol Detection Task

The main task of a communication receiver is to recover the transmitted messages. Channel estimation, studied in the previous subsection, is intended to facilitate the recovery of the unknown symbols. Consequently, we next consider the task of symbol recovery, in which the receiver learns to recover a set of constellation points from its quantized channel output.

As shown in the previous subsection, multivariate complex-values (baseband) can be represented as real vector channels of extended dimensions. Therefore, here we focus on communications over a real-valued MIMO channel. In particular, we consider a BS equipped with $n_{t}=12$ antennas, serving $n_{u}=4$ users. The users transmit i.i.d. binary phase shift keying (BPSK) symbols, represented via the vector $s\in {\left\{-1,1\right\}}^{n_{u}}$. The received signal at the BS, denoted $x\in R^{n_{t}}$, is given by

(8) x=Hs+w,

where $H\in R^{n_{t}\times n_{u}}$ is the channel matrix with entries ${\left(H\right)}_{i,j}=e^{-|i-j|}$, representing spatial exponential decay, and $w\in R^{n_{t}}$ is additive Gaussian noise with zero-mean i.i.d. entries of variance $\sigma_{w}^{2}>0$.

**Figure 6:**
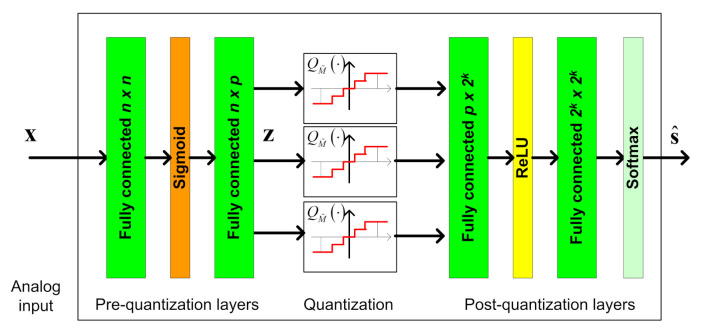
Task-based symbol detector DNN.

Here, the task of the BS is to recover the transmitted symbols vector s from the channel output x, i.e., in this scenario the input dimension is $n=n_{t}$ and the task dimension is $k=n_{u}$. We use a DNN architecture consisting of two fully connected layers in analog and two fully connected layers in digital. As this is a classification task, the output layer is a softmax function with $2^{k}$ probabilities, and the overall network is trained to minimize the cross-entropy loss. An illustration of the DNN structure is depicted in Figure 6. Unlike the scenario considered in the previous subsection, for which the number of quantizers *p* can be set according to the analytical results in [5], here this value was determined based on empirical evaluations. In particular, we use p=⌊kR⌋, resulting in each scalar quantizer using at least n/k=3 bits in the hybrid system.

We compare the achievable bit error rate (BER) of our proposed deep task-based quantizer with soft-to-hard training to using the same architecture with passing gradient training, namely where the quantizers are replaced with additive i.i.d. noise uniformly distributed over the decision regions during training, which is the approach used to train neural networks with intermediate quantization in [25,26,32]. In particular, for the passing gradient method we used a uniform quantization rule over the support [−2,2]. The DNNs are trained using a relatively small training set consisting of t=5000 realizations sampled from the joint distribution of s,x.

The aforementioned data-driven systems are compared to two model-based symbol detectors, which require accurate CSI, i.e., knowledge of H or $\sigma_{w}^{2}$ from which the joint distribution of s and x can be inferred using (8):

1. The MAP rule for recovering s from x *without quantization constraints*, i.e.,
  (9) $${\hat{s}}_{\mathrm{MAP}}≜\underset{s^{\prime }\in {\left\{-1,1\right\}}^{k}}{\arg\max}\mathrm{Pr}\left(s=s^{\prime }|x\right).$$
  The performance of the MAP detector with perfect CSI constitutes a lower bound on the achievable BER of any recovery scheme.
2. The MAP rule for recovering s from a uniformly quantized x with rate *R*, namely
  (10) $${\hat{s}}_{\mathrm{QMAP}}≜\underset{s^{\prime }\in {\left\{-1,1\right\}}^{k}}{\arg\max}\mathrm{Pr}\left(s=s^{\prime }|Q_{\left\lfloor 2^{R}\right\rfloor }^{}\left(x\right)\right),$$
  where $Q_{M^{\prime }}^{}\left(\cdot \right)$ represents the element-wise uniform quantization rule over the interval [−2,2] using $M^{\prime }$ decision regions. The performance of the quantized MAP detector represents the achievable BER when processing is carried out solely in the digital domain, i.e., without using analog processing and/or tunning the quantization mapping in light of the task.

Unlike the detectors based on the MAP rule in (9) and (10), data-driven task-based quantizers do not require CSI, namely no a priori knowledge of H or $\sigma_{w}^{2}$ is used in the detection procedure. Instead, a set of training samples are needed. In order to study the resiliency of our deep task-based quantizer to inaccurate training, we also compute the BER under CSI uncertainty, namely when the training samples are randomized from a joint distribution of s,x in which the entries of the matrix H in (8) are corrupted by additive i.i.d. Gaussian noise, whose variance is 20% the magnitude of the corresponding entry. For comparison, we also evaluate the ber of the MAP rule (9) with the same level of CSI uncertainty. The numerically computed BER values are averaged over 20,000 Monte Carlo simulations.

**Figure 7:**
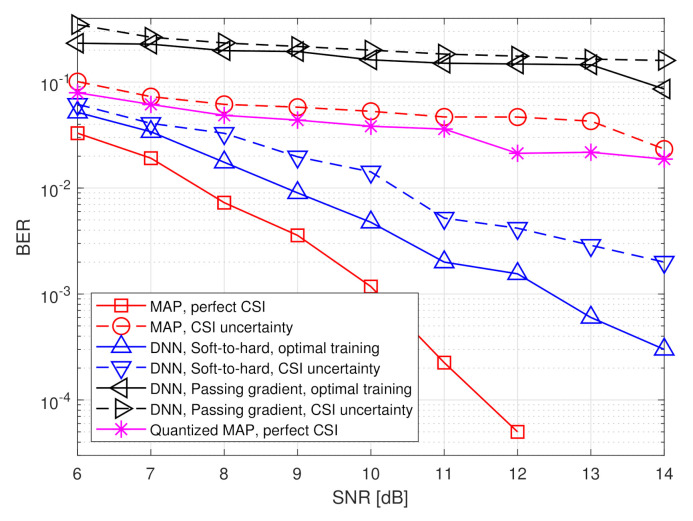
BER versus SNR at rate R=1.

**Figure 8:**
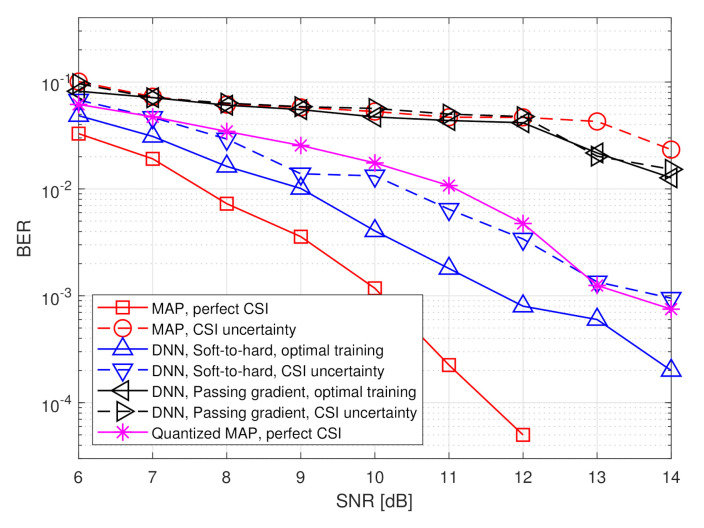
BER versus SNR at rate R=2.

The simulated BER values versus SNR, defined here as $1/\sigma_{w}^{2}$, in the range of [6,14] dB, are depicted in Figure 7 and Figure 8 for quantization rates R=1 and R=2, respectively. Observing Figure 7 and Figure 8, we note that in the presence of accurate CSI, the BER of our deep task-based quantizer is comparable to that achievable using the MAP rule operating without quantization constraints. In particular, while the MAP detector, which is independent of the quantization rate, achieves BER of $10^{-3}$ at SNR of 10 dB, the deep task-based quantizer obtains such BER values at SNRs of 13 and 12 dB, respectively, for quantization rates R=1 and R=2, respectively, namely SNR gaps of 3 and 2 dB. For comparison, the quantized MAP rule, which operates only in the digital domain, does not achieve BER values below $10^{-2}$ at R=1 and requires SNR of over 13 dB to achieve BER of $10^{-3}$ at rate R=2, i.e., with twice the number of bits used by the deep task-based quantizer to achieve the same error rate. This demonstrates the benefit of applying pre-quantization processing in the analog domain, which reduces the dimensionality of the input to the scalar quantizers, thus allowing using more accurate quantization while keeping the semantic information required to classify the symbols from the channel output.

**Figure 9:**
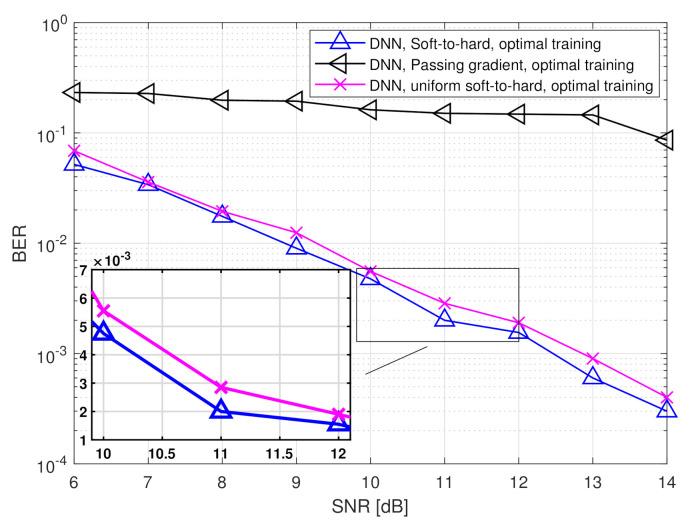
BER versus SNR at rate R=1.

The performance gain of the hybrid DNN architecture stems from the ability to properly model the scalar quantizers during training using our soft-to-hard approach. This model allows to jointly train both the analog and digital DNNs as well as the decision regions of the quantizers, while accurately reflecting the quantization mapping. For comparison, it is observed in Figure 7 and Figure 8 that using the passing gradient approach, i.e., replacing quantization with additive uniformly distributed i.i.d. noise as was done in [25,26,32], leads to substantially deteriorated BER values compared to the proposed soft-to-hard approach. To understand whether the improved gains of soft-to-hard modeling over passing gradient stems from the better approximation of the continuous-to-discrete mapping or from the ability to use non-uniform quantizers, we compare in Figure 9 the performance of the task-based quantizers with soft-to-hard modeling and with passing gradient modeling for the scenario of Figure 7 when using a fixed uniform quantizer with soft-to-hard modeling. In particular, for the uniform soft-to-hard quantizer we used the model in (4) during training with the parameters $\left\{a_{i},b_{i}\right\}$ being fixed to uniform partition of the interval [−2,2], i.e., not optimized during training. It is clearly observed in Figure 9 that most of the gain follows from the usage of an accurate differentiable approximation of the continuous-to-discrete quantization mapping, which allows to train the system in an end-to-end manner while faithfully representing quantization. The gains due to optimizing the decision regions are rather small, indicating that our proposed approach can also lead to substantial improvements when restricted to using uniform scalar quantizers.

The results in Figure 7 and Figure 8 also demonstrate the improved robustness to inaccurate CSI. The performance of the model-based MAP detector is very sensitive to CSI uncertainty, resulting in a notable increase in BER due to the model mismatch. However, the performance of the deep task-based quantizer trained under CSI uncertainty is within an SNR gap of approximately 0.5–2 dB from its achievable performance when trained using accurate CSI. Furthermore, the deep task-based quantizer with CSI uncertainty substantially outperforms the MAP rule without quantization constraints with the same level of uncertainty for all considered scenarios, and outperforms the quantized MAP with accurate CSI for quantization rate of R=1. This demonstrates the gains of using DNNs, with their established generalization properties, for overcoming the sensitivity of model-based approaches to inaccurate knowledge of the underlying parameters.

**Figure 10:**
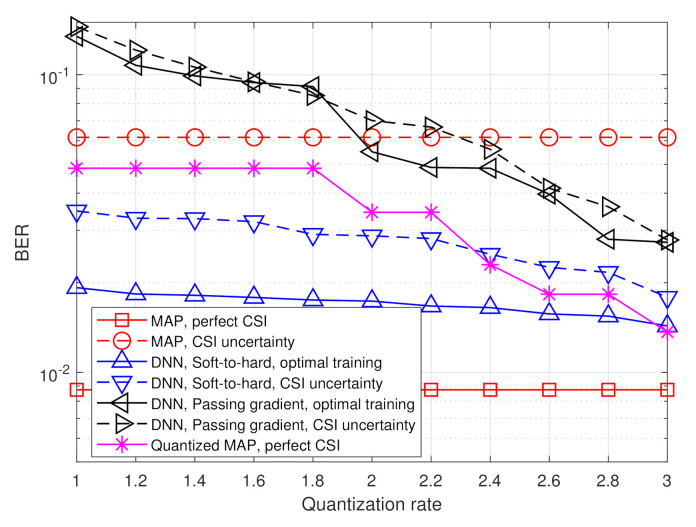
BER versus quantization rate at 8 dB SNR.

**Figure 11:**
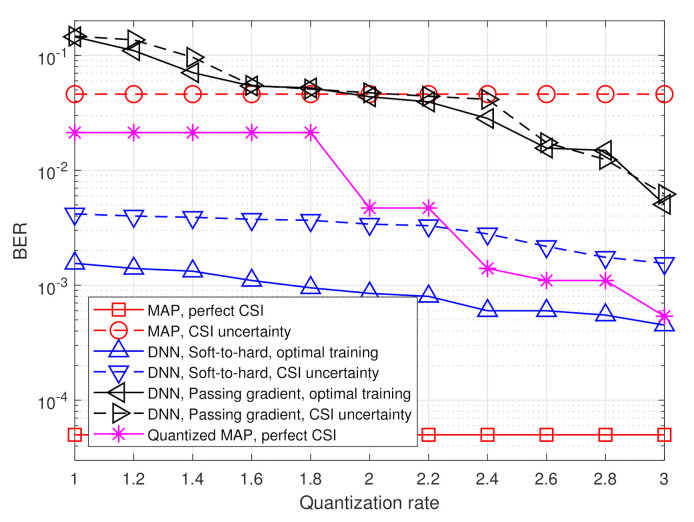
BER versus quantization rate at 12 dB SNR.

Next, we evaluate the BER of the considered quantization systems versus the quantization rate R∈[1,3]. The results are depicted in Figure 10 and Figure 11 for SNR values of 8 dB and 12 dB, respectively. Observing Figure 10 and Figure 11, we note that the gain of the proposed deep task-based quantizer is more dominant when operating with low quantization rates. As the quantization rate approaches three bits per channel input, the BER of applying the MAP in the digital domain via (10) is only within a small gap of the hybrid quantizer with soft-to-hard training. However, for lower quantization rates, as well as in the presence of CSI uncertainty, the proposed deep task-based quantizer maintains its superiority observed in Figure 7 and Figure 8. Furthermore, it is noted that when using the passing gradient training approach, there is a very small gap between the performance achievable with and without CSI uncertainty. This observation is likely due to the fact that when modeling quantization as additive independent noise during training, the network is trained on a mismatched model, regardless of whether the training samples are taken from the same distribution as the test samples. Consequently, such data-driven quantizers operate under some level of uncertainty even when trained using an optimal training set.

Finally, we note that the DNNs used in this subsection were trained using a relatively small training set, consisting of t=5000 samples. This indicates that such architectures can be used to realize an online trainable dynamic ADC, as discussed in Section 3.3.

## 5. Conclusions

In this work we designed a data-driven task-based quantization system, operating with scalar ADCs, using DNNs. We proposed a method for handling the non-differentiability of quantization by approximating its mapping as a smooth function. Our proposed model faithfully represents such continuous-to-discrete mappings while allowing to learn the quantization rule from training. We discussed how this strategy can be used for designing dynamic machine-learning-based ADCs for various tasks. Our numerical results, which considered channel estimation and symbol recovery in bit-constrained MIMO systems, demonstrate that the performance achievable with the proposed deep task-based quantizer is comparable with the fundamental limits for this setup, achievable using optimal vector quantizers. Furthermore, we showed that by using a soft-to-hard approximation of the quantization procedure when training the network in an end-to-end fashion allows the system to be accurately trained with a relatively small training set, and that it notably outperforms the common approach for training DNNs with intermediate quantization.
